# Mental health and job stress of nurses in surgical system: what should we care

**DOI:** 10.1186/s12888-023-05336-0

**Published:** 2023-11-23

**Authors:** Ling Wei, Zhenshan Guo, Xue Zhang, Yanbin Niu, Xiumei Wang, Lifang Ma, Min Luo, Bin Lu

**Affiliations:** 1 Central Operating Department, Shanxi Bethune Hospital, Shanxi Academy of Medical Sciences, Tongji Shanxi Hospital, Third Hospital of Shanxi Medical University, Taiyuan, 030032 China; 2 Hospital-Acquired Infection Control Department, Shanxi Bethune Hospital, Shanxi Academy of Medical Sciences, Tongji Shanxi Hospital, Third Hospital of Shanxi Medical University, Taiyuan, 030032 China; 3 Department of Anesthesiology, Shanxi Bethune Hospital, Shanxi Academy of Medical Sciences, Tongji Shanxi Hospital, Third Hospital of Shanxi Medical University, No. 99 Longcheng Street, Taiyuan, Shanxi Province 030032 China

**Keywords:** Mental health, Job stress, Operating room, Nurses, Care, Nursing

## Abstract

**Background:**

Job stress has significant influence on the mental health of health care providers. The mental health and job stress of operating room nurses remain unclear. This study aimed to evaluate the mental health and job stress of nurses in surgical system in China, to provide evidences for clinical nurse management and care.

**Methods:**

The nurses in the surgical system of our hospital were investigated by questionnaire in December 2022. The general information questionnaire, symptom check list 90 (SCL-90) and nurses’ job stressor scale (NJSS) were used for data collection. Pearson correlation and logistic analysis were conducted to evaluate the related influencing factors.

**Results:**

A total of 171 nurses in surgical system were investigated. The mental health level of nurses in operating room was low. The job pressure of the nurses in the operating room was in the middle level. The nursing profession and work, workload and distribution, working environment and resources, patient care, management and interpersonal relationship were all positively correlated with SCL-90 score of nurses in operating room. Logistic regression analysis indicated that age, year of work experience, professional ranks and titles both are the influencing factors of SCL-90 score and of nurses in operating room.

**Conclusions:**

The mental health of nurses in surgical system is affected by work pressure, ages, working years and professional titles. These factors should be considered in the psychological intervention of nurses in operating room in order to improve the health of clinical nurses.

**Supplementary Information:**

The online version contains supplementary material available at 10.1186/s12888-023-05336-0.

## Introduction

With the continuous development of medical technology and increased workload, the pressure of medical workers is also increasing [1]. It has been reported that nurses’ stress mostly comes from heavy workload, poor working environment, tedious interpersonal relationship processing [2, 3]. Previous study [4] has found that the highest stressors of nurses are nursing workload, management problems and professional conflicts. In China, the nurses are so burdened with work that they often fail to finish their work on schedule during clinical nursing care [5]. Long term job stress can affect disease, fatigue, injury and mental health. Some studies [6, 7] have even shown that individual job stress has an impact on individual psychology, turnover rate and physical and mental health, long-term pressure makes the health status of nurses in a downward trend. Therefore, nurses’ work stress management is very important to nurses’ physical and mental health.

Operating room as an important department of the hospital, in order to improve the quality of operation and ensure the smooth completion of each operation, the demand for medical staff is getting higher and higher, and nurses must have a high degree of attention and sensitive response [8, 9]. The nurses in the operating room are different from the nurses from other clinical departments. When working in the operating room, they must strictly master aseptic operation techniques, good communication skills with doctors and anesthesiologists, and accurately deliver all kinds of equipment, dressings, needle and thread, etc. The pressure they bear is also different from that of nurses in other departments [10, 11]. Currently the mental health and job stress of nurses in surgical system in China remain unclear and needs further investigation. Therefore, this study investigated the professional mental stressors of nurses in the operating room, to understand the job stress and mental health status of nurses in the operating room, to provide evidence for improving the health of nurses in surgical system.

## Methods

This study was a cross-sectional study design, and it was reported accordingly to the Strengthening the Reporting of Observational Studies in Epidemiology (STROBE) statement.

### Ethical consideration

This study was approved by the ethics committee of a tertiary-university hospital in China (approval number: YXLL-2023-096). All the nurses who participated in the survey knowingly and voluntarily agreed to participate in this study. And written informed consents had been obtained from all the included nurses. All the relevant information of the included nurses were processed anonymously, and the relevant data were only used for research purppose.

### Sample size consideration

The sample size was determined according to the number of dimensions of the survey tools [12]. The formula of sample size in this study was: sample size = n × 20 × [1 + (10-15%)]. In this survey, n was 6. In this study, the sample size was calculated as 20 times of the most analytical factors, increasing by 15%, and the calculated sample size was 138 nurses. Considering the validity and integrity of the recovered questionnaire, the sample size of this study should be at least 152 nurses.

### Study population

In this study, the nurses in the operating room of our hospital were investigated by questionnaire in December 2022. The inclusion criteria of the nurses investigated were as following: (1) nurses who worked full-time in the operating room of our hospital; (2) nurses who must obtain nurse qualification certificate; (3) nurses who volunteered to participate in this study. The exclusion criteria of this study were as following: (1) nurses who did not work in the hospital during the survey period (such as maternity leave, going out, etc.); (2) nurses with a history of mental illness. We first asked the nurse whether he had mental illness and the corresponding diagnosis and treatment. In addition, we checked the nurse’s medical treatment records in our hospital with the consent of the nurse to confirm that whether the nurse has a history of mental illness; (3) trainee nurses. (4) nurses who did not want to participate in this study.

### Survey tools

#### The general information questionnaire

This questionnaire was designed by the researchers to collect related information about nurses, including gender, age, education level, year of work experience, professional ranks and titles, and marital status.

#### Symptom check list 90 (SCL-90)

SCL-90 [13] contains 9 symptom factors: Somatization, obsessive-compulsive symptoms, interpersonal sensitivity, depression, anxiety, hostility, phobia, paranoia, psychosis and other 10 factors. A 5-grade scoring system was adopted. 1–5 points represented none, very light, medium, heavy and serious, respectively. The higher the score, the worse the mental health status. The reliability coefficient of Cronbach’s α of SCL-90 was 0.967, which showed good reliability and validity [14].

#### Nurses’ Job stressor scale (NJSS)

The NJSS was compiled by Li et al. [15] was used to analyze the job stress status of nurses. NJSS included 5 dimensions: nursing profession and work, workload and distribution, working environment and resources and patient care and management and interpersonal relationship respectively. The higher the NJSS score, the greater the degree of work stress of nurses. The reliability coefficient of Cronbach’s α of NJSS was 0.98 and the reliability coefficient of each dimension was 0.83–0.95 [16].

### Data collection

In this study, before issuing the questionnaire, after obtaining the support and cooperation of the relevant departments, the questionnaire was numbered and filled in anonymously. And the purpose, significance and method of the investigation were informed before the survey. All the participants allowed to complete it in their own time within three days of issuing the questionnaire. All the survey questionnaires were collected by two investigators and stored in the surgical department of hospital. The survey data were kept confidential and anonymized.

### Statistical analysis

In this study, Epidata3.0 software was used to establish a database for double-entry questionnaire, and consistency monitoring was carried out to ensure the accuracy of data entry. The data were analyzed by SPSS 23.0 statistical software, and the measurement data were expressed by mean ± standard deviation. T-test or one-way analysis of variance of two independent samples was used for comparison among different groups. Pearson correlation analysis was used for potential correlation analysis. We firstly classified the job stress level and mental health status, and then logistics analysis was conducted to identify the potentially influencing factors. The test level of this study was α = 0.05.

## Results

In this study, 176 questionnaires were distributed and collected, and 5 invalid questionnaires were excluded. A total of 171 operating room nurses were investigated. 76.02% nurses were female, the average age was (34.18 ± 10.22) years old, the average years of work experience was (8.94 ± 5.06). The detailed characteristics of included nurses are presented in Table 1.

**Table 1:** The characteristics of included nurses

Characteristic	Cases(n = 171)	Percentage
Gender		
Female	130	76.02%
Male	41	23.98%
Age(y)		
18 ~ 30	56	32.75%
31 ~ 40	79	46.20%
> 40	36	21.05%
Education level		
Junior college degree	61	35.67%
Bachelor degree	108	63.16%
Master degree	2	1.17%
Year of work experience		
< 5	38	22.22%
5 ~ 10	89	52.05%
11 ~ 20	32	18.71%
> 20	12	7.02%
Professional ranks and titles		
Junior nurse	31	18.13%
Senior nurse	74	43.27%
Nurse in charge	45	26.32%
Deputy chief nurse	15	8.77%
Chief nurse	6	3.51%
Marital status		
Unmarried	113	66.08%
Married	58	33.92%

As presented in Table 2, The job pressure of the nurses in the operating room was in the middle level. There were significant differences in the SCL-90 and NJSS scores in nurses with different age, year of work experience and professional ranks and titles (all P < 0.05). There were no statistical differences in the nurses with different gender, educational level and marital status (all P > 0.05). The highest score of nurses’ job stress was (2.19 ± 0.92) on the items of nursing profession and work (Table 3).

**Table 2:** The Symptom check list 90 (SCL-90) and nurses’ job stressor scale (NJSS) score

Characteristic	Cases(n = 171)	SCL-90				NJSS		
		Score	t/F	P		Score	t/F	P
Gender			1.085	0.103			1.294	0.105
Female	143	1.79 ± 0.66				2.43 ± 0.75		
Male	28	1.52 ± 0.48				2.61 ± 0.81		
Age(y)			1.246	0.031			1.146	0.023
18 ~ 30	56	1.75 ± 0.52				2.14 ± 0.87		
31 ~ 40	79	1.87 ± 0.74				2.81 ± 0.93		
> 40	36	1.31 ± 0.62				2.09 ± 0.65		
Education level			1.126	0.098			2.107	0.124
Junior college degree	61	1.72 ± 0.48				2.63 ± 0.86		
Bachelor degree	108	1.67 ± 0.54				2.64 ± 0.92		
Master degree	2	1.75 ± 0.38				2.01 ± 0.88		
Year of work experience			1.107	0.036			1.974	0.042
< 5	38	1.71 ± 0.46				2.03 ± 0.75		
5 ~ 10	89	1.89 ± 0.48				2.86 ± 0.92		
11 ~ 20	32	1.64 ± 0.55				2.84 ± 0.86		
> 20	12	1.37 ± 0.45				2.10 ± 0.81		
Professional ranks and titles			1.182	0.042			1.205	0.016
Junior nurse	31	1.57 ± 0.49				2.10 ± 0.84		
Senior nurse	74	1.78 ± 0.54				2.36 ± 0.71		
Nurse in charge	45	1.77 ± 0.61				2.84 ± 0.85		
Deputy chief nurse	15	1.69 ± 0.58				2.12 ± 0.82		
Chief nurse	6	1.61 ± 0.45				2.10 ± 0.76		
Marital status			1.208	0.117			2.094	0.231
Unmarried	113	1.69 ± 0.56				2.65 ± 0.83		
Married	58	1.78 ± 0.49				2.77 ± 0.84		

**Table 3:** The top 5 scores of job stressors of nurses in operating room

Stressors	NJSS Score
Low wages and other benefits	3.08 ± 0.92
Fewer opportunities for promotion	3.06 ± 0.85
Heavy work burden	3.05 ± 0.91
Not being recognized by patients and their families	3.03 ± 0.89
Worry about mistakes and accidents at clinical nursing care	3.01 ± 0.92

As presented in Table 4, the nursing profession and work (r = 0.449), workload and distribution (r = 0.542), working environment and resources (r = 0.604), patient care (r = 0.581), management and interpersonal relationship (r = 0.537) were all positively correlated with SCL-90 score of nurses in operating room (all P < 0.05).

**Table 4:** Analysis of the correlation between occupational stress and SCL-90 score of nurses in operating room

Items	r	P
Nursing profession and work	0.449	0.012
Workload and distribution	0.542	0.026
Working environment and resources	0.604	0.007
Patient care	0.581	0.018
Management and interpersonal relationship	0.537	0.041

As presented in Table 5, Logistic regression analysis indicated that age (OR = 1.596, 95%CI: 1.224 ~ 1.746), year of work experience (OR = 2.388, 95%CI: 2.103 ~ 2.655), professional ranks and titles (OR = 2.971, 95%CI: 2.703 ~ 3.429) were the influencing factors of SCL-90 score of nurses in operating room (all P < 0.05).

**Table 5:** Logistic regression analysis on the influencing factors of SCL-90 of nurses in operating room

Variables	β	S‾x	OR	95%CI	P
Age	0.113	0.107	1.596	1.224 ~ 1.746	0.034
Year of work experience	0.126	0.112	2.388	2.103 ~ 2.655	0.015
Professional ranks and titles	0.141	0.109	2.971	2.703 ~ 3.429	0.039

As presented in Table 6, Logistic regression analysis indicated that age (OR = 2.066, 95%CI: 1.743 ~ 2.379), year of work experience (OR = 2.129, 95%CI: 1.836 ~ 2.452), professional ranks and titles (OR = 2.575, 95%CI: 2.002 ~ 2.825) were the influencing factors of NJSS score of nurses in operating room (all P < 0.05).

**Table 6:** Logistic regression analysis on the influencing factors of NJSS score of nurses in operating room

Variables	β	S‾x	OR	95%CI	P
Age	0.121	0.113	2.066	1.743 ~ 2.379	0.015
Year of work experience	0.112	0.109	2.129	1.836 ~ 2.452	0.028
Professional ranks and titles	0.134	0.106	2.575	2.002 ~ 2.825	0.041

## Discussions

Operating room nurses have been in a high-pressure environment for a long time, working long hours, high intensity, and exposed to a variety of occupational risk factors, a series of adverse factors make the mental health status of operating room nurses not optimistic [17]. This study has found that the factor scores and total average scores of SCL-90 of nurses in operating room are higher than those of other groups of population, indicating that the mental health status of nurses in operating room is lower than the normal level. The poor mental health status of nurses in the operating room will have a negative impact on their personal and medical behavior. for nurses themselves, poor mental health may affect sleep, and poor sleep quality will in turn affect their mental health, forming a vicious circle and doing harm to their physical and mental health [18, 19]. The poor mental health status of nurses in operating room may cause job burnout and affect the quality of nursing care.

Job satisfaction and burnout in the nursing workforce are global concerns [20]. The work pressure of nurses in operating room mainly comes from the following aspects. First of all, there are the professional and treatment aspects of nursing work. The majority of operating room nurses think that the work pressure is mainly due to lower wages and benefits, lower nursing status and lower promotion opportunities, compared with seniority operating room nurses and anesthesiologists, there are certain differences between their wages and benefits, work status and promotion opportunities [21]. Therefore, operating room nurses lack a sense of self-achievement, and will produce greater psychological pressure. The second is the allocation of workload and time. Previous studies [22, 23] have pointed out that in nursing work, the nursing work in the operating room ranks third in the nursing work pressure of each department, and the hospital considers its own economic benefits and work efficiency, which makes the nurses in the operating room often work overtime for consecutive operations. It has a certain impact on the rest, urination and defecation of nurses in the operating room, and the working state of long-time overload will increase the work pressure. Unfinished care has been shown to be related to staffing, education and working environment, and negatively related to patient outcomes [24]. Besides, the nurses in the operating room are not well recognized and respected by other staff [25, 26], which may have a certain impact on the self-esteem and self-confidence of nurses in the operating room, and virtually increases the pressure of nursing work [27].

Age, working years and professional title are one of the factors affecting the mental health status of nurses in operating room. The mental health status of senior nurses in operating room is worse than that of nurses with other professional titles. There may be two reasons for this phenomenon. First, it comes from the family. The age of the senior nurse is generally between 25 and 30 years old. Women in this age group have to face family issues such as marriage, childbearing and loans. The high cost of the family will also cause them to pay more attention to the issue of wages [28]. Second, it comes from work, senior nurses are the main force in the operating room nurses, they bear heavy tasks and part of the management responsibilities, coupled with the pressure of scientific research and promotion of professional titles, it is easy to produce professional mental pressure [29]. The work schedule in the operating room is usually divided into two shifts, and each shift is arranged by nurses with junior and senior nurses, thus increasing the responsibilities of senior nurses, which further increases their professional mental stress [30]. Therefore, emphasis should be placed on increasing pressure counseling and nursing care for this kind of nurses.

Surgical treatment is inseparable from the close cooperation of the nursing staff in the operating room, and nurses in the operating room need to master more abundant knowledge. The nurses in the operating room not only have to face the intense and heavy work pressure, but also have to face a variety of harmful professional factors and complex interpersonal relationships, which virtually bring greater pressure to the nurses in the operating room [31, 32]. Hospital managers should strengthen the psychological and skill training of nurses, especially pay attention to the cultivation of psychological quality of nurses in operating room, improve their own competitiveness, and master a set of decompression methods suitable for themselves [33]. At the same time, the working time should be arranged reasonably and flexible scheduling should be carried out. High-intensity work will increase nurses’ fatigue and reduce their work efficiency [34], so it is necessary to provide conditions to ensure nurses’ sleep and nutrition. only in this way can a high level of nursing service be provided [35]. Hospital administrators can adopt different methods to improve the mental health level of nurses according to nurses’ age and professional title. For example, they can improve the professional level and comprehensive ability of nurses and nurses by organizing various training activities such as subject lectures [36, 37]. Besides, through performance reform, it’s necessary to optimize salary distribution, form a reasonable assessment system, to increase nurses’ work enthusiasm and income, and conduct regular mental health investigation and consultation on nurses [38, 39]. The nurse managers should grasp the current situation of nurses’ mental health and intervene in time to promote the health of nurses in the operating room [40].

There are some shortcomings in this study that are worth considering. First of all, this study is a single-center cross-sectional survey, although the study sample size is statistically significant, the study sample size is still small, which cannot fully reflect the mental health and stress health status of operating room nurses in other hospitals. Secondly, the characteristics of nurses included in this study are limited, and there may be other factors that affect the health status of nurses. The occupational stress and health status of nurses in the operating room need to be further analyzed by future larger-sample and high-quality researches.

## Conclusion

In conclusion, the nurses in the operating room have high work pressure and low mental health level, and there are great differences in work stress and health level among nurses of different ages, years of work experience and professional titles. Therefore, when carrying out psychological intervention for nurses in operating room, corresponding methods should be adopted according to their different sources of stress and characteristics to reduce job pressure and improve nurses’ health status.

### Electronic supplementary material

Below is the link to the electronic supplementary material.

Supplementary Material 1 - STROBE Checklist

## Data Availability

All data generated or analyzed during this study are included in this published article.

## Abbreviations

SCL-90: Symptom check list 90
NJSS: Nurses’ job stressor scale
STROBE: Strengthening the Reporting of Observational Studies in Epidemiology
